# Exploring Fatigue in Parkinson’s Disease: A Comprehensive Literature Review

**DOI:** 10.7759/cureus.81129

**Published:** 2025-03-25

**Authors:** Jamir Pitton Rissardo, Maleesha Jayasinghe, Masoumeh Rashidi, Fatemeh Rashidi, Hania Moharam, Ibrahim Khalil, Ali Dway, Wael A Elhassan, Mohamed H Elbadawi, Asad UR Rehman, Meryem Bahar, Yixuan Li, Ana Leticia Fornari Caprara, Omesh Prathiraja

**Affiliations:** 1 Neurology, Cooper University Hospital, Camden, USA; 2 Medicine, Nanjing Medical University, Nanjing, CHN; 3 Medicine and Surgery, Nanjing Medical University, Nanjing, CHN; 4 Neurological Surgery, Alexandria Faculty of Medicine, Alexandria, EGY; 5 Faculty of Medicine, AL-andalus university, Tartus, SYR; 6 Faculty of Medicine, University of Khartoum, Khartoum, SDN; 7 Medicine and Surgery, University of khartoum, Khartoum, SDN; 8 Medical Education, Nanjing Medical University, Nanjing, CHN; 9 Biostatistics and Epidemiology, Nanjing Jinling Hexi Campus, Nanjing, CHN; 10 Neurology, Universidade Federal de Santa Maria, Santa Maria, BRA

**Keywords:** apathy, behavioral symptoms, chronic fatigue, depression, excessive daytime sleepiness, fatigue, non-motor symptoms, parkinson's disease, secondary fatigue, sleepiness

## Abstract

Fatigue is one of the most prevalent and debilitating non-motor symptoms of Parkinson’s disease (PD), affecting up to two-thirds of patients and significantly impacting quality of life. This review provides a comprehensive analysis of its complex pathophysiology, prevalence, clinical presentation, assessment methods, and current management strategies. Fatigue in PD is linked to dysfunction in dopaminergic and non-dopaminergic pathways, neuroinflammation, genetic predispositions, and metabolic dysregulation. Reported prevalence rates range from 36% to 60%, highlighting the need for standardized assessment tools and a universally accepted definition. Clinically, fatigue is characterized by an overwhelming and abnormal sense of exhaustion, often preceding motor symptoms by years. Distinguishing it from sleepiness, apathy, and depression is crucial for accurate diagnosis and treatment. Assessment primarily relies on patient-reported scales, such as the Fatigue Severity Scale and the Parkinson’s Disease Fatigue Scale. Management includes both pharmacological and non-pharmacological approaches, with medications like methylphenidate and rasagiline showing potential, alongside physical exercise, cognitive behavioral therapy, and sleep disorder management. Further research is essential to unravel the underlying mechanisms, identify objective biomarkers, and develop more effective, targeted treatments for this burdensome symptom.

## Introduction and background

Parkinson's disease (PD) is characterized by the progressive degeneration of dopaminergic neurons in the substantia nigra pars compacta (SNpc), leading to motor impairments. The number of PD cases has increased significantly from 1990 to 2019, likely due to aging populations and improved diagnostic methods [1]. PD is a neurodegenerative disease that presents with a wide range of symptoms in both the motor and nonmotor domains. The disease is most commonly associated with motor symptoms such as bradykinesia, rigidity, tremors, and gait disturbances. However, nonmotor symptoms, such as cognitive, mood, autonomic, and sleep disturbances, are also common and can significantly impact a person's quality of life. These nonmotor symptoms can be caused by factors such as neurotransmitter imbalances, neuroinflammation, and the widespread distribution of Lewy bodies [2].

Fatigue is a diverse and intricate symptom that is typified by an intense sense of tiredness, depletion, or low energy. It might show itself as a physical, mental, or emotional condition that affects a person's capacity to go about their everyday life normally [3]. Fatigue associated with PD is also known as “Park fatigue.” This unique symptom needs special consideration in diagnosis and treatment, as it can seriously impair a person's quality of life [4]. Fatigue is a prevalent non-motor symptom of PD, affecting half of the patients, according to a meta-analysis of 44 studies including 7,427 individuals. This research emphasizes how crucial it is to identify and treat fatigue in treating PD [5].

In 2016, Kluger et al. established diagnostic criteria for fatigue associated with Parkinson's disease. These criteria require patients to report a significant decline in energy levels or an excessive perception of effort that is disproportionate to their level of activity. Symptoms must persist for most of the day, occurring daily or nearly every day over the past month. Routine activities of daily life, involving minimal or no exertion, can trigger these symptoms. The patient's type, intensity, or duration of activities can be severely affected by the symptoms, often requiring prolonged periods of rest for alleviation of the effects. Symptoms can be induced by social interactions, cognitive tasks, or any tasks that require concentration for prolonged periods. People with Parkinson's disease, in a bid to avoid exacerbating their symptoms, are likely to avoid strenuous activities; however, even mild to moderate levels of activity can worsen the symptoms over a period of time. Symptoms may be sudden-onset in nature or follow a predictable pattern, occurring at specific times of the day. As a result, the patient may experience significant distress or impairment in social, occupational, or other important areas of life due to fatigue. There is evidence from the patient's history and physical examination suggesting that fatigue is likely a consequence of Parkinson’s Disease. Furthermore, the symptoms are not primarily caused by other conditions, such as comorbid psychiatric disorders (e.g., depression), sleep disorders (e.g., obstructive sleep apnea), or other medical conditions (e.g., anemia or congestive heart failure) [6].

In PD, depression is a prevalent comorbidity that can cause fatigue on its own. Effective care necessitates determining the underlying cause of fatigue, as depression-induced fatigue cannot be treated with the same approaches as PD-related fatigue. Understanding the many causes of weariness enables the development of customized therapies to enhance patients' quality of life [7]. Friedman et al. followed 51 patients with PD for 9 years, finding that 21 continued to experience significant fatigue. This suggests that fatigue is a persistent problem for many PD patients, even over a long period [8]. When compared to PD patients with mild fatigue, those with severe fatigue showed a reduced proportion of REM sleep and a higher likelihood of REM sleep behavior disorder (RBD). Even after controlling for many other variables, these components remained independent predictors of fatigue. Furthermore, even after controlling for factors such as age, sex, length, body mass index, illness severity, depression, anxiety, and other sleep disorders, the percentage of REM sleep and the amount of REM sleep without atonia continued to be linked to fatigue severity ratings [9]. High levels of anxiety and depression, as determined by the hospital Anxiety and Depression Scale (HADS) depression and anxiety subscales and the Non-Motor Symptoms Scale (NMSS) for PD assessing mood domain, were substantially correlated with fatigue [10]. A lower standard of living, a higher chance of falls, and damaged relationships are just a few of the effects that can drastically affect their day-to-day existence. It is important to address these many facets in order to enhance the general well-being and quality of life of people with PD [11].

Certain medications used to treat PD, including dopamine agonists and anticholinergics, may contribute to fatigue as a side effect, likely due to their impact on neurotransmitter systems and brain circuits that regulate energy and motivation. Careful monitoring and adjustment of medication regimens or exploring alternative medication or non-pharmacological interventions may be necessary to minimize medication-induced fatigue in PD patients [12]. Compared to patients with lower LED (levodopa equivalent dosage), those with higher LED exhibited much more intense weariness. So, increasing levodopa dosages could play a role in PD-related fatigue severity. When changing a patient's levodopa dose for PD, clinicians should be aware of this possible adverse effect [13]. Even though understanding and treating fatigue in PD has advanced significantly, more work must be done before the condition is effectively managed. Methylphenidate and other medications provide some optimism, but further studies are needed to verify their long-term effectiveness and investigate other possible pharmacological treatments. While there is currently no clear evidence linking exercise to fatigue, leading an active lifestyle is recommended for general health reasons and its ability to alleviate other symptoms of PD. Establishing a commonly acknowledged definition of fatigue in PD is still a crucial first step since it will open the door to more precise diagnostic instruments and focused therapies. We can enable people with PD to recover their energy and quality of life by investigating various management techniques and further researching the intricate nature of tiredness [14].

This review aims to provide a comprehensive understanding of fatigue in PD, encompassing its pathophysiology, prevalence, clinical manifestations, assessment, and management strategies. We aim to delve into the underlying mechanisms of fatigue in PD, exploring the roles of dopaminergic and non-dopaminergic pathways, inflammation, genetic factors, and neuroendocrine dysregulation. Investigate the prevalence and epidemiology of fatigue in PD, providing insights into its impact on the PD population. Describe the clinical manifestations of fatigue in PD, including its symptoms, impact on quality of life, and differentiation from other PD symptoms. Discuss the various assessment tools used to evaluate fatigue in PD and identify factors contributing to its severity.

## Review

Pathophysiology

Overview of Fatigue in Neurological Disorders

Fatigue is one of the non-motor symptoms that usually accompany many neurological conditions such as PD, multiple sclerosis, myasthenia gravis, stroke, and various autonomic dysfunctions. It significantly affects the quality of life [15]. Different classifications of fatigue have been introduced in the literature. Some papers classify fatigue into physical and pathological fatigue. The first type results from prolonged, intense activities while the chronic form appears regardless of the intensity of the activities [16,17]. Fatigue can also be classified as physical fatigue, which represents the physical exhaustion from exercise, and mental fatigue, which affects cognitive abilities [18,19]. One of the most widely used classifications in the literature is the categorization of fatigue into central and peripheral fatigue.

Central fatigue refers to continuous tiredness from a chronic condition while the peripheral definition represents the inability to sustain muscle contraction [20]. Multiple studies have been conducted in the context of sleep in neurological disorders. A new classification has been introduced due to the discovered association between fatigue and some non-motor symptoms, such as mood and sleep disorders, which classify fatigue into primary and secondary types [15,21]. Fatigue is considered secondary if it is accompanied by a mood or sleep disorder while isolated fatigue is considered primary [22]. Fatigue has also been found to correlate with the severity of PD in multiple studies [10,21]. The association between fatigue and sleep disorders in PD has been studied across a wide range of research. It is believed that PD can cause circadian dysfunction, and the opposite sequence can also occur, with PD resulting from circadian problems [23]. A study conducted by Skorvanek et al. reported a significantly higher prevalence of fatigue among the secondary fatigue group compared to the primary fatigue group, demonstrating a possible correlation between mood and sleep disorders with fatigue [22]. Fatigued PD patients tend to present with more sleep problems, more cognitive problems, pain, urinary symptoms, depression, anxiety, and subsequent poor quality of life [24,25]. Some studies suggest that fatigue can be one of the important predictors of sleep problems [26], while other studies in neurological disorders propose a separate pathophysiological mechanism for sleep disturbances in PD by addressing central nervous system abnormalities and circadian rhythm disorders [27]. Therefore, more studies need to be conducted to determine whether sleep disturbances are merely a consequence of fatigue in patients with PD. Multiple mechanisms and pathophysiologies have been introduced in the literature regarding fatigue in PD. These mechanisms vary widely in anatomical and physiological pathophysiology.

Neurobiological mechanisms

Dopaminergic Pathways

Dopamine is one of the most common catecholamines synthesized in the central nervous system (CNS), and it is synthesized in the substantia nigra - affected in PD - and the ventral tegmental area [28]. The role of dopamine in the pathophysiology of fatigue remains controversial. Some studies have found positive effects of dopamine and reported less fatigue progression among patients with PD who took levodopa [12]. Additionally, two studies on rasagiline (a monoamine oxidase inhibitor drug that increases dopamine concentration in the striatum) reported its benefits in decreasing the fatigue scale [29,30]. Moreover, another clinical trial was conducted using methylphenidate, which blocks the reuptake of dopamine and norepinephrine. The trial concluded that the drug significantly lowered the fatigue scale [31]. On the contrary, multiple studies have found no effect of dopamine agonists on fatigue [32,33].

The most important of the opposite studies is a meta-analysis conducted in 2023 on pharmacological and non-pharmacological interventions in patients with PD. Due to a lack of studies in the pharmacological part, only modafinil, which inhibits the reuptake of dopamine and increases extracellular dopamine, was included in the meta-analysis. The study revealed a non-significant effect of the drug on patients' fatigue with PD (Standardized Mean Difference (SMD) = -0.21, 95% Confidence Interval (CI) -0.74 to 0.31, p = 0.43) [34].

Several structural and functional imaging studies have been conducted on multiple neurological disorders. These studies showed abnormalities in the prefrontal cortex, which receives dopamine projections, and the frontal lobe in fatigued patients [35]. Moreover, striatal infarcts were found to be associated with fatigue in stroke patients [36]. In addition, reduced perfusion to the frontal lobe was significantly associated with fatigue in patients with PD [37].

Non-dopaminergic Systems

Another neurotransmitter hypothesized to be related to fatigue in PD is serotonin. The main reason for the hypothesis is that fatigue is one of the symptoms of depression, and it is treated by increasing serotonin levels [38]. Serotonin pathways are also believed to be affected by PD as Lewy bodies accumulate in the median raphe nuclei, which contain serotonergic neurons [39]. A study by Pavesa et al. reported a significant reduction in serotonin transporter binding in the striatum and thalamus of fatigued patients with PD, which supports the role of serotonin in fatigue. ​In this study, the researchers specifically selected patients without a history of depression or sleep disturbances. This careful selection was intended to isolate fatigue as an independent symptom of Parkinson's disease, minimizing potential confounding factors [40]. Moreover, another study by Yamamoto et al. on chronic fatigue reported a reduction in serotonin transporter density using radioactive material (11CMcN5652), which may also be observed in patients with PD [41]. However, a study by Pauletti et al. mentioned some concerning findings due to a significant reduction in serotonergic central tone between patients with PD and healthy controls but no difference between patients with PD with fatigue and those without it. They used loudness dependence of auditory evoked potentials to measure the central tone, and they concluded that fatigue may result from serotonin/dopamine imbalance rather than serotonin deficiency alone. More research is required to identify the intercorrelation between serotonin and dopamine in causing fatigue [42].

Other than dopamine and serotonin, other neurotransmitters and chemicals have also been examined for their effect on fatigue in patients with PD. Caffeine, an adenosine antagonist, was reduced in patients with PD complaining of fatigue, but the effect was insignificant. This requires more studies on the involvement of adenosine [43]. Moreover, glutamate was also found to precipitate fatigue in these patients. A clinical trial conducted in 2022 and a review in 2021 reflected the effect of safinamide (which inhibits abnormal glutamate release) in eliminating and preventing fatigue episodes. However, this may be due to an additional mechanism of the drug, which also affects monoamine oxidase (MAO) and causes a subsequent elevation in dopamine concentration, so further investigations are required [44]. Few studies have studied the effect of gamma-aminobutyric acid (GABA)ergic dysfunction on fatigue, but not in PD, such as the study by Versace et al., who reported an increase in fatigue due to a reduction in GABAergic inhibition in the primary motor cortex [45].

Role of inflammation

Neuroinflammation was found to play a role in the pathophysiology of neurological diseases and even contribute to the progression of PD [46]. The role of inflammation has been examined in many neurological and muscular diseases before such as multiple sclerosis and PD [47]. The role of inflammation in fatigue among patients with PD has been indirectly discovered by measuring inflammatory markers. According to a review conducted in 2019, higher serum interleukin-6 (IL-6), interleukin-1 receptor antagonist (IL-1-Ra), soluble interleukin-2 receptor (sIL-2R), and vascular cell adhesion molecule-1 (VCAM-1) were found to be associated with higher fatigue levels among patients with PD. Surprisingly, the review reported a lower level of fatigue associated with higher serum uric acid, an indicator of oxidative stress [48]. This can be justified as serum uric acid was reported not to have a significant correlation with fatigue scores in multiple sclerosis [47]. A study by Lindqvist et al. reported the elevation of another inflammatory marker among fatigued patients whose C-reactive protein was elevated (p = 0.008) [49]. However, soluble tumor necrosis factor receptor (sTNFR), a tumor necrosis factor-alpha (TNF-α) antagonist, was not different between fatigued patients with PD and non-fatigued patients [50]. While TNF-α was found to have a positive association with the fatigue scale [51]. Different mechanisms have been presented for the role of these cytokines and chemokines in causing fatigue, such as microglial activation, recruitment of leukocytes, direct effects on neurotransmission, and effects on the hypothalamic-pituitary axis [52]. Another suggested mechanism is the elevation in pro-inflammatory cytokines due to chronic inflammation. These elevated levels disrupt the blood-brain barrier by interacting with its endothelium. These cytokines work in the brain to prevent the astrocytes from re-uptaking glutamate, which can lead to an increase in glutamate concentration and, subsequently, the precipitation of fatigue [53].

Genetic factors

Genetic factors also affect the fatigue mechanism, as several mutations have been linked with fatigue in patients with PD. One of these mutations is the heterozygous glucocerebrosidase mutation, which was reported to be associated with more fatigue in PD patients (p = 0.001) [54]. Mutation in this enzyme also increases the risk of PD itself, as glucosylceramide can accumulate in different visceral organs, including the central nervous system (CNS) [55]. Another gene studied is the leucine-rich repeat kinase 2 (LRRK2) G2385R variant, a known PD risk factor [56]. People with the LRRK2 G2385R variant were found to have significantly higher levels of fatigue than non-carriers (p = 0.002) [57]. Interestingly, the carriers of this gene were also found to have an increased prevalence of autonomic dysfunction and sleep disorders [58]. Moreover, the absence of the Parkin gene, as in PD, was found to promote mitochondrial impairment, a known source of cell energy, and could be a possible mechanism of fatigue [59]. Another important genetic factor is a mutation in the gene-producing alpha-synuclein, which was found to cause familial PD [60]. A hypothesis that illustrates the ability of elevation in alpha-synuclein to cause fatigue in PD patients is presented. First, abnormal aggregation of this protein can occur due to inflammation or mutations in amino acid sequences. Then, a conformational change shifts the molecule to insoluble beta sheets [53,61]. Alpha-synuclein then activates toll-like receptors (TLR4) on microglial cells, releasing many pro-inflammatory cytokines. These cytokines, as mentioned previously, can inhibit the reuptake of glutamate by astrocytes, promoting fatigue in PD patients [53]. What supports this mechanism is the growing evidence of a significant association between elevated levels of cerebrospinal fluid (CSF) alpha-synuclein and fatigue in PD patients [51,62].

Neuroendocrine and metabolic dysregulation

The hypothalamic-pituitary axis has also been shown to contribute to the pathophysiology of fatigue in PD. This axis has vast connections to wide brain areas, such as the basal ganglia, amygdala, thalamus, and frontal cortex, which may precipitate the pathology in the case of axis dysfunction [63]. Several neurological studies have addressed the endocrine system's role in fatigue induction. A study conducted by Gottschalk et al. reported significantly higher levels of adrenocorticotropic hormone (ACTH) in multiple sclerosis patients with fatigue [64]. On the contrary, Schaefer et al. reported decreased cortisol levels among fatigued patients with CNS infections [65]. This illustrates the need for more studies to understand the role of ACTH and cortisol in fatigue induction in patients with PD. Other hormones, such as testosterone, have also been tested in patients with fatigue but have not been found to correlate with fatigue [66].

From a muscular system point of view, multiple mechanisms have been studied. In the case of the absence of the Parkin gene, electron transport chain genes are down-regulated. This disrupts oxidative phosphorylation and increases oxidative stress. The subsequent mechanism leads to mitochondrial dysfunction and damage, which may affect the cells' energy levels [59]. On the contrary, a study conducted by Stevens-Lapsley et al. concluded that less fatigue in patients with PD was due to a reduction in central activation, preventing muscle overload and the subsequent metabolic fatigue [67]. The musculoskeletal system's role remains controversial, as studies show a variable range of findings. In a meta-analysis conducted in 2023, physical exercise was found to be a good treatment for fatigue, which is inconsistent with the lack of energy and central activation mentioned in other studies [34].

Other mechanisms

The autonomic system has also been linked to fatigue, as fatigue was found to be correlated with autonomic dysfunction [15]. In addition, the management of autonomic symptoms and depression in PD is quite helpful in fatigue treatment [68]. Multiple brain areas, such as the frontal, temporal, and parietal regions, have been found to correlate with fatigue [48]. Imaging studies in patients with PD reflected increased activity in areas other than the basal ganglia, such as the premotor cortex and cerebellum, as a compensatory mechanism. These areas lead to differences in corticospinal excitability, and further studies must address their role in fatigue [69].

Cardiac sympathetic innervation plays a crucial role in modulating cardiovascular responses to physical exertion, including changes in heart contractility and chronotropic regulation. Studies analyzing cardiac metaiodobenzylguanidine (MIBG) heart-to-mediastinum uptake in fatigued and non-fatigued individuals with PD identified a possible link between fatigue and cardiac sympathetic denervation [70]. While typically asymptomatic in PD, this denervation may impair autonomic cardiovascular regulation, potentially leading to reduced cardiac contractility during exercise. This dysfunction could contribute to exercise intolerance, manifesting as shortness of breath and an increased perception of fatigue. Moreover, the dysregulation of catecholaminergic signaling in PD may further exacerbate these symptoms, limiting the body's ability to adapt to exertional demands, and even impairing cognition [68]. Understanding the relationship between fatigue and cardiovascular dysautonomia in PD could provide new insights into therapeutic strategies aimed at mitigating fatigue-related disability.

Prevalence and epidemiology

One of the most common non-motor symptoms of PD that affects a sizable percentage of patients is fatigue. Numerous studies have revealed prevalence percentages ranging from 36.8% to 59.46%, underscoring the diversity of research populations and evaluation techniques. Several studies included in this table provide valuable insights into the prevalence of fatigue in PD (Table 1).

**Table 1:** Prevalence of fatigue in Parkinson's disease PD: Parkinson’s Disease; PFS: Parkinson Fatigue Scale; FSS: Fatigue Severity Scale; IPD: Idiopathic Parkinson’s Disease; SD: Standard Deviation; NA: Not Available

Reference	Sample size	Age (mean)	Sex (Male)	Prevalence of fatigue	Comments
Siciliano et al. (2017) [11]	81 consecutive de novo PD patients	65.73 (SD: 8.26)	52	15%(n=12) of patients reported distressing fatigue (defined as a PFS score ≥ 8)	15% of patients with early, de novo PD reported distressing fatigue.
Friedman et al. (2001) [8]	26	NA	NA	At the initial assessment, 42% of the patients reported fatigue as one of their three most disabling symptoms. At the follow-up assessment, 50% of the patients reported fatigue as their most disabling symptom, and 62% reported it as one of their three most disabling symptoms.	Fatigue is a common and often disabling symptom, affecting a significant proportion of PD patients.
Diaconu et al. (2024) [25]	131 PD patients and 131 age- and sex-matched healthy controls	NA	NA	In PD patients: 38.16% reported fatigue based on the Chalder fatigue scale. 46.54% reported fatigue based on the PFS. In healthy controls: 26.71% reported fatigue based on the Chalder fatigue scale.	Highlight the importance of recognizing and addressing fatigue in PD management.
Souza et al. (2024) [71]	80	53.55 years (SD: 10.8)	80	Mean FSS score was 36.97 ± 16.45, indicating a moderate level of fatigue in the sample	The study focuses on men with PD, a population that is often understudied in terms of sexual health.
Minibajeva et al. (2023) [72]	43	65.21 years (SD 8.9)	20	95.3%	Non-motor symptoms, which are often overlooked but significantly impact the quality of life of PD patients
Zhou et al. (2023) [73]	2100	60.47 years	1048	36.8%	Routinely assess for fatigue in PD patients. Fatigue is associated with increased disease severity and progression. Clinicians should consider the potential impact of fatigue on quality of life when managing PD patients.
Nassif et al. (2022) [74]	53	Non-fatigued group: 64.75 years (SD: 7.23). Fatigued group: 65.71 years (SD: 8.72)	Non-fatigued group: 68.75%. Fatigued group: 66.67%.	39.62%	High prevalence of fatigue (39.62%) in PD patients, which is consistent with other studies. The study found that fatigue was associated with worse quality of life, which is an important finding.
Güler et al. (2022) [75]	9887 (118 with IPD (idiopathic PD))	78.6 years	58.4% of the PD patients were male	46.8%	In this study, all the numbers are for the 188 patients with IPD
Ineichen et al. (2021) [76]	337	69.3	38.3%	Significant fatigue (FSS total score ≥ 4): 40.3%. Severe fatigue (FSS total score ≥ 5): 17.8%	
Siciliano et al. (2020) [77]	55	64.7	NA	At baseline: 22% using the Parkinson Fatigue Scale (PFS) cut-off. At 1-year follow-up: 38% using the PFS cut-off.	Fatigue affected 22% of the population at baseline, and increased over time to 38% at 1-year follow-up.
Siciliano et al. (2018) [5]	7427	NA	NA	50%	Systematic review and meta-analysis
Fu et al. (2016) [78]	222	NA	NA	59.46%	Fatigue is influenced by multiple factors beyond motor symptoms. While dopaminergic treatment can be beneficial for some patients, addressing sleep disturbances and depression may be crucial for effectively managing fatigue in PD.

These studies show that around 36% to 60% of people with PD experience tiredness. It is probable that variations in research populations, evaluation techniques, and definitions of weariness account for the fluctuations in prevalence rates. It is important to note that fatigue can significantly impact the quality of life of PD patients, affecting their daily activities, social interactions, and overall well-being. Therefore, recognizing and addressing fatigue is crucial for comprehensive PD management.

Mukadam et al. studied how the COVID-19 pandemic affected subjective cognition and social functioning in people with PD. A longitudinal analysis of 123 patients revealed that declines in these areas were linked to increased anxiety, fatigue, and motor symptoms. Fatigue, in particular, was a significant factor in worsening quality of life during the pandemic. Additionally, subjective cognitive decline correlated with depression and social functioning decline. Women reported a greater perceived impact of COVID-19, while in men, personal COVID-19 experience was associated with cognitive decline [79].

Clinical manifestations

PD patients describe their fatigue as a “feeling of abnormal and overwhelming tiredness and lack of energy that is distinct both qualitatively and quantitatively from normal tiredness [80].” Fatigue was considered to be related to the pathology of PD. It has a pre-motor feature, as it can occur early in the course of the disease, preceding the onset of other motor symptoms related to PD [81]. Moreover, a 16-year United Kingdom study indicated that experiencing fatigue five years prior to a PD diagnosis elevated the relative risk of developing the disease to 1.56 [82]. In early PD, non-motor symptoms, such as fatigue, may be more disabling than motor symptoms [83]. ​In the study by Miwa et al., fatigue was found to significantly impair the quality of life (QOL) in patients with PD. The researchers evaluated 46 outpatients with PD, excluding those with cognitive impairments. They discovered that 48% of these patients experienced fatigue. Further analysis revealed that fatigue was closely associated with reduced QOL and higher apathy scores. Interestingly, depression and excessive daytime sleepiness did not show a significant correlation with fatigue in this study [84].

In the context of PD, fatigue should be precisely defined, as there are several confounding symptoms. To address this, a unifying taxonomy has been proposed by Kluger et al. to define fatigue in clinical and research contexts [16]. This taxonomy is based on five main criteria: 1) Differentiating fatigue from related phenomena such as sleepiness; 2) Recognizing the perception of fatigue, which is a sensation of exhaustion or increased effort, as phenomenologically distinct from performance fatigue, which is a decline in performance caused by continuous effort on a demanding task; 3) Distinguishing clinically significant (pathological) fatigue from normal physiological fatigue; 4) Identifying potential causal factors based on their function (e.g., homeostatic, psychological) or neuroanatomical location (e.g., central, peripheral); 5) Specifying the domain(s) of performance affected such as motor or cognitive functions.

Fatigue in PD can be classified into two types: peripheral fatigue, which is a measurable condition that occurs when a muscle loses strength after repeated contractions, and central fatigue, which is a subjective feeling that cannot yet be objectively measured [85].

Fatigue represents a considerably debilitating experience for PD patients. Patients with PD who experience fatigue are more likely to develop apathy [86], and this association is also linked to other important factors such as depression [30], cognitive dysfunction, and reduced quality of life [87]. Patients experiencing fatigue had a longer duration of their illness and more severe motor symptoms, except for tremors. They also showed more depressive symptoms and experienced greater sleep disturbances than those without fatigue. Additionally, the severity of sleep disturbances was found to be an independent factor contributing to fatigue [78]. Due to fatigue, some patients were forced to decrease their working hours, in addition to reducing their participation in social activities [88]. ​In the study by Cochrane et al., fatigue and apathy were prevalent in both PD and multiple sclerosis (MS) patients, with significant correlations between the two symptoms in both disorders. Specifically, 64% of PD and 74% of MS patients experienced severe fatigue, while apathy was present in 57% of PD and 52% of MS patients [89].

PD fatigue showed a pronounced impact on quality of life. A prospective study demonstrated a significant association between PD fatigue and overall health-related quality of life (HRQOL) [90]. Moreover, another study reported fatigue as the most important non-motor symptom affecting health-related quality of life (HRQOL) [91], a finding that is supported by another study [92]. The latter study revealed the presence of a relationship between fatigue and depression, as well as fatigue and sleep disturbances among idiopathic Parkinson's disease (IPD) patients. Additionally, depression and sleep disturbances were more prevalent in the IPD group with fatigue compared to the IPD group without fatigue [92]. One of the challenges in addressing and managing fatigue in PD is the high prevalence of potentially confounding symptoms such as daytime sleepiness, apathy, and depression [87]. Like depression, fatigue is often linked with daytime sleepiness and sleep disorders. However, it can be distinguished by the fact that sleep does not provide restoration, and it may also occur in PD patients who have normal sleep patterns [93]. A recent case-control study revealed that PD patients with fatigue had fewer night sleep hours compared to those without fatigue. Moreover, patients with fatigue experienced more pronounced pain, anxiety, and depression. Additionally, restless legs syndrome was more prominent in patients with fatigue compared to those without fatigue (International Restless Legs Syndrome Study Group score 5.61 ± 7.98 vs. 2.49 ± 6.26, p = 0.013). At the same time, higher worry and less happiness were recorded in patients with fatigue. On the other hand, this study also showed the fatigue impact on cognitive capacity, as lower scores in the Mini-Mental State Examination (MMSE) and Montreal Cognitive Assessment (MoCA) scales were detected among PD patients with fatigue, indicating greater cognitive dysfunction compared to patients without fatigue [25].

Assessment of fatigue in Parkinson's disease

Since fatigue is a subjective symptom, patient self-reported questionnaires remain the primary method for either diagnosing or measuring fatigue severity [94]. The Fatigue Severity Scale (FSS) is a nine-item scale used to assess the physical aspects of fatigue and its impact on quality of life (QOL) [95]. It evaluates how fatigue affects routine activities such as exercise, physical functioning, work duties, responsibilities, and family and social life. Each item is expressed as a statement in the form of a concise declaration that is evaluated using a Likert scale ranging from one to seven. Grade 1 represents a complete disagreement while Grade seven represents a complete agreement. The FSS total score, which is the average of the nine items' scores, indicates the severity of fatigue, with higher scores indicating a more severe level of fatigue. This scale is recommended by the International Movement Disorder Society and has been validated in different languages [96].

**Figure 1:**
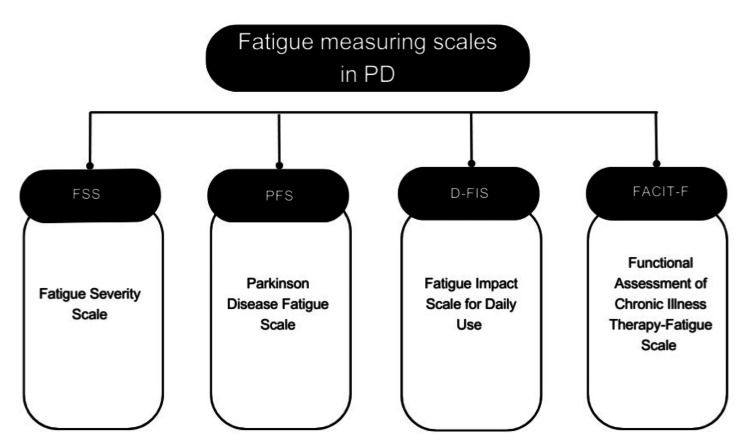
Summary of common scales used to assess fatigue in Parkinson's disease PD: Parkinson's Disease; FSS: Fatigue Severity Scale; PFS: Parkinson's Fatigue Scale; D-FIS: Fatigue Impact Scale for Daily Use; FACIT-F: Functional Assessment of Chronic Illness Therapy-Fatigue Original figure created by the authors

In 2005, Brown et al. developed a scale known as the Parkinson’s Disease Fatigue Scale (PFS) and confirmed its validity and reliability [80]. This scale is intended to be used mainly for PD patients. The PFS is a 16-item self-reported questionnaire designed to evaluate the physical aspects of fatigue in patients with PD. The PFS offers two scoring methods. The original method features response options ranging from one (strongly disagree) to five (strongly agree), with the total PFS-16 score ranging from one to five, obtained by dividing the sum of all item scores by 16. The binary scoring method, on the other hand, transforms item responses into 1s and 0s, with “agree” and “strongly agree” being scored as 1s, and all other responses as 0s. This method yields a total score between 0 and 16, with 16 representing higher levels of fatigue. Another scale called the Fatigue Impact Scale for Daily Use (D-FIS) is an 8-item scale developed to measure the subjective daily experience of fatigue [97]. Additionally, another generic fatigue questionnaire has been used to assess fatigue in PD; it is called the Functional Assessment of Chronic Illness Therapy-Fatigue Scale (FACIT-F) [98]. Figure 1 summarizes the different scales used to assess fatigue in PD.

Fatigue is a prevalent and debilitating non-motor symptom in PD, requiring accurate assessment tools for both clinical and research purposes. Several fatigue scales are available, each differing in structure, scoring system, and clinical utility [99]. The Fatigue Severity Scale (FSS) and Parkinson’s Fatigue Scale (PFS) are among the most widely recommended by the Movement Disorders Society (MDS) for screening and severity assessment. The FSS, consisting of 9 items scored on a 7-point Likert scale, evaluates the impact of fatigue over a two-week period, with a total score range of 9 to 63 [100]. The PFS, specifically designed for PD, includes 16 items scored from 16 to 80 and focuses on the presence and impact of fatigue over the same timeframe [101]. Both scales have established cutoff values and are considered reliable measures for assessing fatigue burden in PD. Other scales, such as the Fatigue Assessment Inventory (FAI) and Functional Assessment of Chronic Illness Therapy-Fatigue Scale (FACIT-F), offer broader evaluations but with some limitations. The FAI, with 29 items scored on a 7-point scale, includes a definition of fatigue and assesses symptoms over two weeks, though it is only suggested by the MDS for screening. The FACIT-F, comprising 13 items on a 5-point scale, assesses fatigue over one week with a total score range of zero to 52. While it has a defined cutoff for clinical significance, it is not specific to PD, which may limit its sensitivity in this population [102]. The Multidimensional Fatigue Inventory (MFI), a 20-item scale evaluating fatigue across multiple domains, lacks established cutoff values and has not been strongly recommended for Parkinson’s disease-related fatigue assessment. Additional fatigue scales, such as the Fatigue Severity Inventory (FSI), Daily Fatigue Impact Scale (D-FIS), and Fatigue Visual Analog Scale (FVAS), are also used but with varying degrees of validation in PD. The D-FIS, an 8-item scale assessing fatigue on a single day, is suggested for use but lacks defined cutoff values. The FSI, with 33 items scored on a 7-point scale, and the FVAS, a simple visual scale, are listed by the MDS but not strongly recommended. The Clinical Global Impression Scale (CGIS), which provides a variable and subjective assessment, is also listed but lacks a standardized scoring system [103]. Future studies are needed to refine these tools and determine the most effective scale for capturing PD-related fatigue and its impact on daily functioning.

Factors contributing to fatigue in Parkinson’s disease

Although fatigue in PD is primarily caused by the disease's pathological disturbances, several other factors may contribute to the onset and increasing severity of fatigue in PD. In general, strong associations between levels of depression, anxiety, and fatigue in PD were demonstrated in several studies. Fatigue in PD was associated with the severity of depressive symptoms. The association between depression and fatigue has been addressed in several studies. On the other hand, anxiety has been shown to have an association with fatigue in PD. In general, strong associations between levels of depression, anxiety, and fatigue in PD were demonstrated in several studies [5,21,104-106].

Poor sleep is indicated as a major contributor to fatigue in PD [107]. For this reason, careful attention to nighttime sleep is required. The association between sleep disturbances and fatigue has been addressed in several studies [108]. Additionally, a recent longitudinal study revealed that poor baseline sleep was associated with greater fatigue and depression after one year [109]. At the same time, a cross-sectional study reported that anxiety is a significant predictor of poor sleep quality [110]. The correlation between fatigue severity and the Non-Motor Symptoms Scale (NMSS) was significant [111]. This was evident in the presence and frequency of disturbances in the affective sphere (anhedonia, loss of interest, apathy, and anxiety). Additionally, sleep disorders, such as daytime sleepiness and sleep difficulties, were also strongly associated with fatigue severity. Furthermore, gastrointestinal and sexual dysfunctions were linked to the severity of fatigue [111]. These findings support the idea that both the affective sphere and sleep disorders are closely related to the development of fatigue. This is consistent with Metta et al., who found a connection between fatigue and sleep dysfunction, as well as anxiety and depression [10]. A recent study reported that individuals diagnosed with de novo PD and experiencing distressing fatigue displayed elevated levels of sleepiness, depression, anxiety, and apathy [11]. Such findings suggested that the presence of distressing fatigue, as assessed by the Parkinson’s Fatigue Scale (PFS), is linked to a higher load of non-motor symptoms [111]. Further analysis in the same study using logistic regression revealed that only sleepiness, cognitive apathy, and episodic anxiety were the key independent factors with the greatest explanatory power in relation to distressing fatigue. Kwon et al.’s study explores the relationship between motor and non-motor symptoms in patients with de novo PD. Among 105 analyzed patients, those with non-tremor-dominant PD exhibited more severe fatigue and dysautonomia compared to tremor-dominant patients while other non-motor symptoms, such as cognition, depression, and anxiety, showed no significant differences. Further analysis revealed that postural instability and gait difficulty were significantly associated with fatigue (r = 0.3659, p = 0.0002), suggesting a potential link between motor impairment and fatigue severity. These findings highlight the impact of motor subtypes on fatigue, emphasizing the need for targeted interventions in PD management [112].

In terms of the relationship between fatigue and cognitive apathy, several studies reported the association between fatigue and cognitive apathy in PD [11,89,104]. Moreover, fatigue was demonstrated to be associated with poor decision-making [113]. On the other hand, fatigue is associated with the severity of motor symptoms, as indicated by the Unified Parkinson's Disease Rating Scale Part III (UPDRS III), as well as with disease disability [15]. A prospective longitudinal study by Alves et al. demonstrated a correlation between fatigue and PD severity [114]. A recently conducted study indicated that non-motor symptoms tend to have a greater impact on fatigue than motor symptoms [115]. The study showed that there is a link between fatigue and higher Body Mass Index(BMI) scores, increased levels of anxiety and depression, lower daily equivalent levodopa dose (LEDD), and more severe non-motor symptoms of daily life activities (DLAs).

Differences in non-motor symptoms have been observed between genders for PD [116]. Few studies investigated the relationship between fatigue and gender in PD, but these studies are contradictory. Some studies indicated that gender was associated with fatigue [117]. At the same time, a recent review of 22 studies revealed that only one study showed less fatigue in males [5]. Additionally, females with concurrent chronic illness in the general population have been described as experiencing higher levels of fatigue [118]. Therefore, the relationship between fatigue and gender is more likely influenced by general factors rather than PD-specific factors. Therefore, this issue needs to be further investigated.

In terms of the relationship between fatigue and medication, longitudinal studies on fatigue in early PD have produced contradictory findings regarding the relationship between dopamine and fatigue. While Ongre et al. demonstrated that de novo PD patients using dopamine agonists experienced lower levels of fatigue compared to those using levodopa [119], Ou et al. found higher fatigue prevalence in patients with higher LEDD at the two and three-year follow-up marks [120].

Overlap of fatigue with other conditions

Fatigue is a prevalent non-motor symptom in PD, affecting approximately 44.2% of patients, as compared to 18% in healthy elderly individuals [121]. This symptom often coexists with conditions such as depression, dementia, and sleep disturbances. However, studies indicate that even PD patients without these comorbidities report significant levels of fatigue, suggesting that fatigue may be an independent manifestation of PD rather than solely a consequence of overlapping conditions.

Moreover, there is a notable overlap between fatigue and excessive daytime sleepiness in PD patients. Research demonstrates that while both symptoms are prevalent, they exhibit different correlations with disease characteristics. For instance, excessive daytime sleepiness is more closely associated with disease duration and the type of dopaminergic treatment, whereas fatigue shows a stronger correlation with depressive symptoms. This distinction underscores the complex interplay between various non-motor symptoms in PD and highlights the necessity for comprehensive assessment and tailored management strategies for affected individuals [122].

Sleep Disorders

Fatigue and sleep disturbances are prevalent non-motor symptoms in PD, significantly impacting patients' quality of life. Over 75% of individuals with PD experience sleep-related issues, including insomnia, excessive daytime sleepiness (EDS), restless legs syndrome (RLS), and rapid eye movement (REM) sleep behavior disorder (RBD). Fatigue often correlates with these sleep disturbances, creating a cyclical pattern where poor nighttime sleep exacerbates daytime fatigue, and vice versa. Addressing sleep disorders through proper management and treatment is crucial in alleviating fatigue and enhancing the overall well-being of PD patients [9].

Depressive Disorders

Fatigue and depression are common and often overlapping non-motor symptoms in PD, contributing to significant impairment in daily functioning. Research suggests that depression affects around 36% of PD patients while fatigue is reported by nearly 40%, with both conditions frequently coexisting [123]. This overlap complicates diagnosis and treatment, as fatigue may be influenced by both psychological and neurological factors inherent to PD. Studies also indicate that individuals with both symptoms experience a more severe disease burden, including increased disability and diminished quality of life.

The relationship between fatigue and depression in PD is complex, with evidence suggesting a bidirectional influence. Depression can intensify perceptions of fatigue while persistent fatigue may contribute to the development or worsening of depressive symptoms. Additionally, neurobiological mechanisms, including disruptions in dopaminergic and serotonergic pathways, are implicated in both conditions, highlighting the need for targeted therapeutic strategies. Addressing fatigue in PD requires a multidimensional approach, incorporating pharmacological treatments, behavioral interventions, and lifestyle modifications to improve overall patient well-being.

Cognitive Deﬁcits

Fatigue and cognitive deficits are prevalent non-motor symptoms in PD, often presenting concurrently and significantly impacting patients' quality of life. Research indicates that cognitive impairments, particularly in executive functions, may be associated with cognitive fatigue, as patients struggle with tasks requiring sustained mental effort. Additionally, attention deficits have been linked to fatigue in PD, suggesting that difficulties in maintaining focus may contribute to the perception of fatigue [124].

The interplay between fatigue and cognitive deficits in PD is complex, with evidence pointing toward a bidirectional relationship. Cognitive impairments can exacerbate feelings of fatigue while persistent fatigue may further impair cognitive performance, creating a vicious cycle that hinders daily functioning. Understanding this relationship is crucial for developing effective interventions aimed at alleviating both fatigue and cognitive deficits in PD patients [125].

Deep brain stimulation and Parkinson’s disease fatigue

Bilateral subthalamic deep brain stimulation (STN-DBS), a widely recognized treatment for motor symptoms in PD, has also shown potential benefits for non-motor symptoms. However, its effects on fatigue remain insufficiently studied, with small-scale investigations yielding inconsistent results [126].

The exact mechanisms driving this improvement remain uncertain but are likely linked to STN-DBS modulation of the basal ganglia-thalamo-cortical and limbic circuits [127]. Nevertheless, the studies assessing deep brain stimulation in fatigue had small sample sizes, lacked a control group, and the absence of a Parkinson’s disease-specific fatigue scale, which limits the generalizability of the results. Future research using larger cohorts and Parkinson’s disease-specific fatigue assessments is needed to clarify the effects of STN-DBS and identify potential predictors of treatment response.

A recent retrospective cross-sectional study reviewed the medical records of 50 individuals with PD who underwent STN-DBS, with an average follow-up of 1.98 ± 1.36 years. Fatigue was evaluated using the Non-Motor Symptoms Scale, revealing a significant median improvement of 41.5%. However, no baseline or follow-up factors were identified as significant predictors of fatigue improvement [128]. These findings suggest that STN-DBS may help alleviate PD-related fatigue.

Management

**Figure 2:**
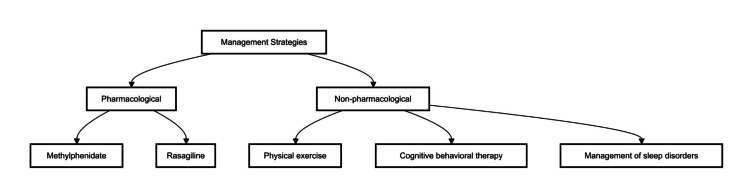
Comprehensive management strategies for addressing fatigue in Parkinson’s disease, bifurcated into pharmacological and non-pharmacological categories Pharmacological management includes methylphenidate, a stimulant medication showing promise in alleviating fatigue, and rasagiline, a monoamine oxidase-b inhibitor that may help reduce fatigue symptoms. Non-pharmacological management encompasses physical exercise, which is recommended to improve overall energy levels, cognitive behavioral therapy (CBT) to address behavioral and cognitive aspects, and proper diagnosis and treatment of sleep disorders, which can significantly impact fatigue levels. The figure underscores the multifaceted approach required to manage fatigue in PD effectively, combining both pharmacological and non-pharmacological treatments to enhance patient quality of life. Original figure created by the authors

Managing fatigue in PD requires a multifaceted approach tailored to address both its physiological and psychological contributors. Non-pharmacological strategies, such as structured exercise programs, CBT, and sleep optimization, have shown benefits in reducing fatigue severity. Additionally, lifestyle modifications, including maintaining a balanced diet, managing stress, and ensuring proper hydration, can help improve energy levels. Pharmacological options, such as dopaminergic medications, stimulants, or antidepressants, may be considered in cases where fatigue significantly impacts daily functioning. However, individualized treatment plans are essential, as responses to therapy vary among patients. A comprehensive management approach that combines behavioral, pharmacological, and rehabilitative strategies can enhance overall well-being and quality of life for individuals with PD (Figure 2).

Pharmacological management

Fatigue is a prevalent non-motor symptom in PD, yet its pharmacological management remains challenging due to limited and inconclusive evidence. Levodopa, the cornerstone of PD motor symptom treatment, does not consistently alleviate fatigue, although some studies suggest it may slow its progression. This inconsistency underscores the need for alternative therapeutic options.

Stimulant medications, such as modafinil and methylphenidate, have been explored for their potential to reduce fatigue in PD patients. Methylphenidate is a dopamine transporter blocker (blocks the reuptake of dopamine and norepinephrine in the presynaptic neurons). A placebo-controlled study showed a statistically significant reduction in the Fatigue Severity Scale [31]. However, clinical trials have yielded mixed results, with some individuals reporting benefits while others experience minimal improvement. Notably, these stimulants are not currently approved for treating PD-related fatigue, highlighting the necessity for further rigorous studies to establish their efficacy and safety in this context.

Another pharmacological agent, rasagiline, an MAO-B inhibitor, has been investigated for its effects on fatigue in PD. It proved to have a statistically significant reduction in the Parkinson's Fatigue Scale [30]. While some studies indicate that rasagiline may reduce fatigue symptoms, the observed effect sizes are generally small and may not translate into clinically meaningful improvements. Consequently, more comprehensive research is required to determine the true benefit of rasagiline in managing PD-related fatigue.

Intrajejunal levodopa infusion (IJLI) has been investigated for its effects on non-motor symptoms, including fatigue, in PD patients. A prospective open-label observational study involving 22 advanced PD patients demonstrated significant improvements in six of the nine domains of the Non-Motor Symptoms Scale (NMSS), notably including the sleep/fatigue domain, following six months of IJLI therapy [129]. These findings suggest that IJLI may effectively alleviate fatigue in PD patients, potentially enhancing their overall quality of life. However, further large-scale, randomized controlled trials are necessary to confirm these benefits and to better understand the long-term impact of IJLI on fatigue management in PD.

Doxepin, a tricyclic antidepressant, has been investigated for its potential to alleviate fatigue in PD. A study by Rios Romenets et al. found that doxepin reduced fatigue severity and its impact on activities of daily living in PD patients [130]. However, the authors noted that these findings require confirmation through larger, high-quality randomized controlled trials. Regarding dopaminergic medications, research indicates that levodopa may improve physical fatigue in PD patients. A study by Lou et al. demonstrated that levodopa administration led to significant improvements in physical fatigue during finger-tapping and force-generation tasks [12]. However, the authors emphasized the need for further studies to confirm these results and understand the underlying mechanisms. Despite these potential pharmacological interventions, there is currently no definitive treatment for PD-related fatigue.

Non-pharmacological interventions

A study conducted in 2014 reported that high-intensity exercise in PD patients resulted in a significant improvement in fatigue by 17% [131]. Moreover, a clinical trial showed that aerobic exercise accounted for a 0.5-point reduction in fatigue severity scale in PD [132]. Adding to this, a recent meta-analysis investigating physical exercise as an intervention to treat fatigue in PD reported a standardized mean difference (SMD) of -0.37 (95% CI - 0.69‐ 0.05) when compared to the placebo group [34], demonstrating a significant small effect of physical exercise on fatigue. At the same time, a randomized-controlled trial showed that home-based treadmill training is effective in reducing fatigue in patients with mild PD [133]. In the latter study, treadmill training was associated with a 1.2-point reduction in the 7-point fatigue scale. Despite this, further research is still required to examine the effectiveness of exercise in improving fatigue in PD.

Another possible intervention is cognitive behavioral therapy (CBT). It has been demonstrated that CBT, a non-pharmacological approach to alter psychological states and ameliorate psychological issues, is beneficial for mental illnesses [134]. A recent meta-analysis investigating the efficacy of CBT on mood disorders, sleep, and fatigue in PD showed a significant impact of CBT on depression, anxiety, and sleep disorders, yet no significant effect on fatigue [135]. However, since fatigue is linked to several contributing factors, especially neuropsychiatric ones, as has been discussed before, CBT remains a potential choice, and its efficacy warrants further investigation.

The impact of sleep quality on fatigue progression is pivotal, as a positive correlation was indicated between sleep disorders and fatigue severity in PD [10]. Moreover, it was found that PD patients with severe sleep disturbances are more likely to experience fatigue [78]. Excessive Daytime Sleepiness (EDS), defined as undesired sleepiness during waking hours, has been found to influence fatigue in PD [136]. Addressing the factors associated with poor sleep quality in PD, such as depression, represents a beneficial approach to improving sleep quality. [137]. On a related note, a recent clinical trial showed that acupuncture therapy is beneficial in improving the quality of sleep in PD and thus quality of life [138]. The Tan et al. study aimed to systematically assess the effectiveness of traditional Chinese exercises in managing neuropsychiatric symptoms in individuals with Parkinson's disease. While some benefits were observed, the analysis revealed no statistically significant improvements in fatigue-related measures [139].

Specialist recommendations and future studies

Many biomarkers have been suggested to indicate fatigue in PD patients. As previously mentioned, neuroinflammation plays a role in fatigue in PD patients. Thus, multiple inflammatory markers can be used to detect fatigue, such as interleukin (IL)-6, IL-1Ra, sIL-2R, and VCAM-1 [48]. A study by Crichton and the team in traumatic brain injury also reported that IL-8 can serve as a possible indicator of fatigue in these patients [140]. This illustrates the importance of further studies on IL-8 and other inflammatory markers to identify fatigue in PD.

A recent exploratory study examined the cerebrospinal fluid of individuals with PD who had low versus high fatigue scores. Using label-free liquid chromatography and tandem mass spectrometry, researchers identified 20 differentially expressed proteins. Supervised partial least squares discriminant analysis (PLS-DA) revealed that these proteins were associated with innate immunity, cellular stress responses, and sickness behavior, suggesting that these biological processes contribute to fatigue in PD. While the study’s small sample size limited the ability to control for confounding variables, the findings highlight the need for larger-scale investigations to further clarify the molecular mechanisms underlying PD-related fatigue [141].

The latest systematic reviews on PD patients' fatigue (2018 and 2023) included only cross-sectional studies, with a significant lack of exploration of longitudinal studies on fatigue [5,142]. Another systematic review conducted in 2015 concluded that there are no clear recommendations for managing fatigue in PD patients. Interestingly, it also highlighted the importance of further studies on the behavioral or cognitive aspects of fatigue in PD patients, which was also supported by another study in 2018 [143]. Moreover, most of the conducted studies focused on fatigue as one group with the same contributing factors. For this reason, Skorvanek et al. recommended studying fatigue in PD patients into two separate groups: primary and secondary fatigue, due to differences in their clinical and psychosocial factors [22]. Even the recent meta-analysis on interventions for fatigue in PD has shortcomings, as it only covered one pharmacological option in the analysis, which is modafinil. Additionally, the authors suggested further studies on the efficacy of physical exercises in the treatment of fatigue. This calls for more studies to cover a wide range of pharmacological and non-pharmacological options [34].

Kluger et al. suggested some recommendations in 2015 to improve clinical research in this area such as distinguishing fatigue from other related disorders (apathy, depression, etc.) [6]. This was also suggested by Siciliano and colleagues, who additionally strengthened the importance of identifying fatigue biomarkers and establishing a proper fatigue management protocol [5]. Kluger et al. continued with further recommendations, such as differentiating between subjective and objective fatigue, and specifying the domains and causal factors affected by either type of fatigue [6]. In addition to areas of intervention, further exploration of the pathophysiology of PD is still required. Wasson et al. conducted a study on fatigue in older adults. They noticed that higher mental fatigability is associated with smaller basal ganglia and limbic systems. The same basal ganglia play a central role in the pathophysiology of PD. That is why this hypothesis needs further studies in the PD population [144].

## Conclusions

Fatigue in Parkinson's disease presents one of the greatest challenges to patients and clinicians alike, and its complexity characterizes it as a highly multifactorial phenomenon. This means that fatigue is not the result of a simple consequence of the illness but rather an interaction among neurobiological, inflammatory, genetic, and psychosocial factors. The hallmark dopaminergic pathway dysfunction of Parkinson's disease must also necessarily be causally involved since studies have shown some improvement with levodopa and dopamine agonists. However, variable results and the limited efficacy of dopaminergic therapies suggest that non-dopaminergic systems also contribute to fatigue, which is related to serotonin, norepinephrine, and adenosine.

The role of chronic inflammation is substantiated by a growing body of evidence in Parkinson's disease, where raised inflammatory markers, such as interleukin-6 and tumor necrosis factor-alpha, have been observed in association with greater severity of fatigue. Genetic predispositions to heightened fatigue risk in Parkinson's disease patients are also attributed to genes such as leucine-rich repeat kinase 2 and glucocerebrosidase beta mutations. These factors are further complicated by psychosocial issues, which include depression, anxiety, sleep disturbances, and medication side effects, creating a feedback loop that further exacerbates fatigue and negatively impacts overall well-being and quality of life. The causative factors are so intricately interwoven that not only the motor symptoms but, importantly, the non-motor burden of this highly debilitating disease make compelling arguments for a multifaceted approach to assessment and management.
